# Mesothelin Virus-Like Particle Immunization Controls Pancreatic Cancer Growth through CD8^+^ T Cell Induction and Reduction in the Frequency of CD4^+^foxp3^+^ICOS^−^ Regulatory T Cells

**DOI:** 10.1371/journal.pone.0068303

**Published:** 2013-07-09

**Authors:** Sheng Zhang, Lin-Kin Yong, Dali Li, Rafael Cubas, Changyi Chen, Qizhi Yao

**Affiliations:** 1 Molecular Surgeon Research Center, Michael E. DeBakey Department of Surgery, Baylor College of Medicine, Houston, Texas, United States of America; 2 Department of Molecular Virology and Microbiology, Baylor College of Medicine, Houston, Texas, United States of America; 3 Department of Pathology and Immunology, Baylor College of Medicine, Houston, Texas, United States of America; Mie University Graduate School of Medicine, Japan

## Abstract

Our previous study has shown that mesothelin (MSLN) is a potential immunotherapeutic target for pancreatic cancer. Here, we further studied the immunogenicity of chimeric murine MSLN-virus-like particles (mMSLN-VLPs), their ability to break tolerance to mMSLN, a self-antigen, and deciphered the mechanism of immune responses elicited by mMSLN-VLP immunization using a pancreatic cancer (PC) mouse model. In addition to what we have found with xenogeneic human MSLN-VLP (hMSLN-VLP), mMSLN-VLP immunization was able to break the tolerance to intrinsic MSLN and mount mMSLN-specific, cytotoxic CD8^+^ T cells which led to a significant reduction in tumor volume and prolonged survival in an orthotopic PC mouse model. Furthermore, CD4^+^foxp3^+^ regulatory T cells (Tregs) were progressively decreased in both spleen and tumor tissues following mMSLN-VLP immunization and this was at least partly due to elevated levels of IL-6 production from activated plasmocytoid dendritic cell (pDC)-like cells following mMSLN-VLP immunization. Moreover, mMSLN-VLP treatment mainly reduced the frequency of the CD4^+^foxp3^+^ICOS^−^ Treg subset. However, mMSLN-VLP induced IL-6 production also increased ICOSL expression on pDC-like cells which supported the proliferation of immunosuppressive CD4^+^foxp3^+^ICOS^+^ Treg cells. This study reveals that mMSLN-VLP immunization is capable of controlling PC progression by effectively mounting an immune response against mMSLN, a tumor self-antigen, and altering the immunosuppressive tumor microenvironment via activation of pDCs-like cells and reduction in the frequency of CD4^+^foxp3^+^ICOS^−^ Treg cells. However, combination therapies will likely need to be used in order to target residual CD4^+^foxp3^+^ICOS^+^ Treg cells.

## Introduction

Pancreatic cancer remains a devastating, highly lethal disease even with the current scientific advancements. This disease represents an enormous challenge to clinicians and scientists because it is naturally resistant to various forms of treatments [1]. Therefore, there is an urgent need to develop novel therapies for pancreatic cancer. Among the most recent therapeutic approaches, cancer vaccines have shown some promising results for disease control [2]. Many have been reported to be promising in inducing *in vivo* tumor regression [XPATH ERROR: unknown variable "start2".], [4]. However, the mechanism of how tumor vaccines can successfully control tumor progression is still unclear.

Tumor-specific cytotoxic T lymphocyte (CTL) induction has been shown to be essential for the eradication of cancer cells by effective anti-tumor vaccines 5,6 since CTLs can be specific to a particular antigen expressed by tumor cells. In addition, the role of regulatory T cells (Tregs) in anti-tumor immunity has been greatly studied and elucidated [7]. Treg cells identified as CD4^+^CD25^+^foxp3^+^ represent the main inhibitory lymphocyte population [8], [9]. Removal of Treg cells by *in vivo* administration of an anti-CD25 antibody has been shown to abrogate immune suppression, limit tumor growth, and promote tumor rejection in mice [10]. The co-stimulatory molecule ICOS is one of the regulatory proteins expressed on CD4^+^CD25^+^foxp3^+^ Tregs [11]. The expansion of Tregs can be linked to ICOS signaling, which also participates in the development of antigen-specific Tregs [12]. A recent report has shown that foxp3^+^ Tregs have two distinct subsets with different biological functions based on ICOS expression. One of these subsets, CD4^+^foxp3^+^ICOS^+^ Tregs, secretes IL-10 and TGF-β which suppresses dendritic cells (DCs) and CD4^+^ helper T cells. The other subset, CD4^+^foxp3^+^ICOS^−^ Tregs, only produce TGF-β [13]. A study has also shown that murine Tregs contain hyperproliferative and death-prone subsets with differential ICOS expression [14]. However, the response of these two Treg subsets to tumor immunotherapeutic vaccination has not been investigated.

The induction and generation of foxp3^+^ Tregs is associated with DC function [15]. Although both conventional DCs (cDCs, myeloid DC) and plasmacytoid DCs (pDCs) can interact with foxp3^+^ Treg cells, only pDCs are reported to have an ability to prime CD4^+^foxp3^+^ICOS^+^ Tregs [13]. pDCs are known as a primary DC subset in anti-viral immune responses [16]. Upon activation and maturation, they secrete large levels of type I interferons which activate other innate immune cells like cDC and NK cells and bridge adaptive immune cells like T cells and B cells [17]. pDCs not only have the capacity of presenting MHC-II epitopes in order to activate CD4^+^ T cells, but can also cross-present MHC-I epitopes to expand CD8^+^ T cells [18]. The induction of CD4^+^foxp3^+^ICOS^+^ Tregs by pDCs, but not cDC, is coupled to ICOS ligand expression on pDCs [13].

The effectiveness of cancer immunotherapeutic approaches relies heavily on the immunogenicity of the targeted tumor-associated antigen. Mesothelin (MSLN), is a membrane glycoprotein normally expressed on mesothelial cells, but is over-expressed in most pancreatic cancers [19]–[21]. The limited level of MSLN expression in normal cells makes it a proper therapeutic target for cancer vaccines [22]. Among the various vaccine approaches that carry and present tumor antigens to the host, virus-like particles (VLPs) may be the most suitable for inducing both humoral and cellular immune responses. As an analog of viruses, chimeric VLPs can be considered incompetent viruses, which have the complete virus structure and viral protein components to stimulate strong cellular responses without viral nucleic acids. VLPs can induce preventive or therapeutic immunity not only against tumor-related virus infection which can lead to tumorigenesis [23], [24], but they can also inhibit tumor growth by specifically targeting tumor associated antigens [25]. We have previously shown that chimeric hMSLN-VLPs can effectively decrease tumor growth through a reduction in the frequency of CD4^+^foxp3^+^ Tregs in an orthotopic pancreatic cancer mouse model [21]. In this study, we further evaluated whether self-antigen mMSLN incorporated on VLPs can break the tolerance to mMSLN, mount adaptive immune responses and control tumor progression. To further understand the mechanism of action by which VLP immunization can achieve tumor regression, we also explored properties of DC induction by mMSLN-VLP and cytokines involved in the suppression of CD4^+^foxp3^+^ Treg cells.

## Results

### mMSLN-VLP Immunization Inhibits Pancreatic Cancer Growth and Promotes Survival in Tumor-bearing Mice

**Figure 1:**
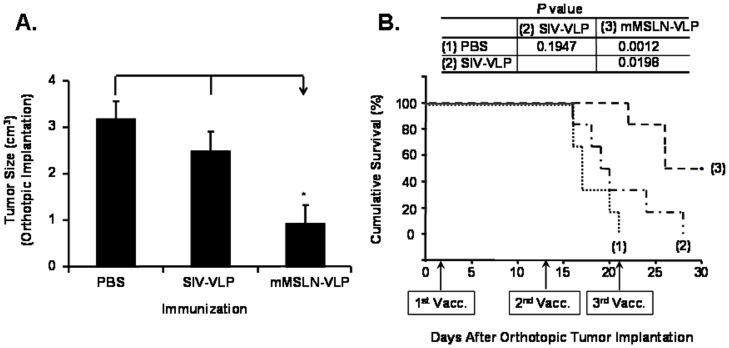
Chimeric mMSLN-VLP vaccination reduced tumor burden and prolonged survival in a Panc02-bearing orthotopic pancreatic cancer mouse model. Panc02 cells (7×10^5^) were orthotopically implanted at pancreas of 8-week old C57BL/6 mice at day 0. Three immunizations at days 3, 13, and 21 were applied by i.p. route with different VLPs. The mice were divided in three groups which immunized with either 100 µl of PBS, or 100 µg/100 µl of SIV-VLP, or 100 µg/100 µl of MSLN-VLP. **A).** Reduced tumor burden in mMSLN-VLPs immunized groups. Tumor size between each group was statistically compared by student t-test. *p<0.05. **B).** Prolonged survival of mMSLN-VLPs immunized mice groups Kaplan-Meier Survival Analysis. *P* values were calculated and listed on the figure. These were representative data selected from four independent experiments with A) five or B) at least 9 mice per group.

In our previous report, we demonstrated that immunization with human MSLN-VLP could effectively control tumor growth in a mouse model which led to the enhancement of cytotoxic immune responses against pancreatic cancer cells through a reduction in the frequency of Treg cells [21]. Since human and mouse MSLN has only about 60% homology, the strong immune response may result from a xenogeneic response to human MSLN. However, whether immunization with mMSLN-VLP can break the tolerance and induce immune responses to mMSLN, a self-antigen, in the context of a VLP presentation, and whether mMSLN-VLP has a similar effect in eliciting an anti-tumor response by modulating Tregs has not been explored. To evaluate the immunotherapeutic efficacy of mMSLN-VLP immunization, animals were immunized with either mMSLN-VLP or SIV VLP as a control for the VLP “backbone” or PBS in tumor-bearing mice. As shown in Fig. 1A, mMSLN-VLP immunization significantly reduced tumor volume (0.92±0.39) compared with the two control groups (2.49±0.43 in SIV-VLP and 3.18±0.38 in PBS control group) (*p*<0.05). There was no statistical difference in tumor burden between the PBS and SIV-VLP immunization groups. Furthermore, we found that all mice treated with PBS or SIV-VLP had 1 ml to 4 ml ascites or bloody ascites in the abdominal cavity. There was less fluid (<1 ml) in the mMSLN-VLP vaccinated group. In addition, as shown in Fig. 1B, with SIV-VLP immunization, there was a slightly prolonged survival yet not statistically significant when compared to the PBS control group (*p* = 0.1947). On the contrary, mMSLN-VLP immunization significantly prolonged the survival of tumor-bearing mice when compared to the PBS and SIV-VLP groups (*p* = 0.0012 and *p* = 0.0198, respectively). Furthermore, more than 50% of the mMSLN-VLP immunized mice were still alive on day 30.

### mMSLN-VLP Immunization Activates CD8^+^ T cells and Generates MSLN-Specific Functional CD8^+^ T cells

**Figure 2:**
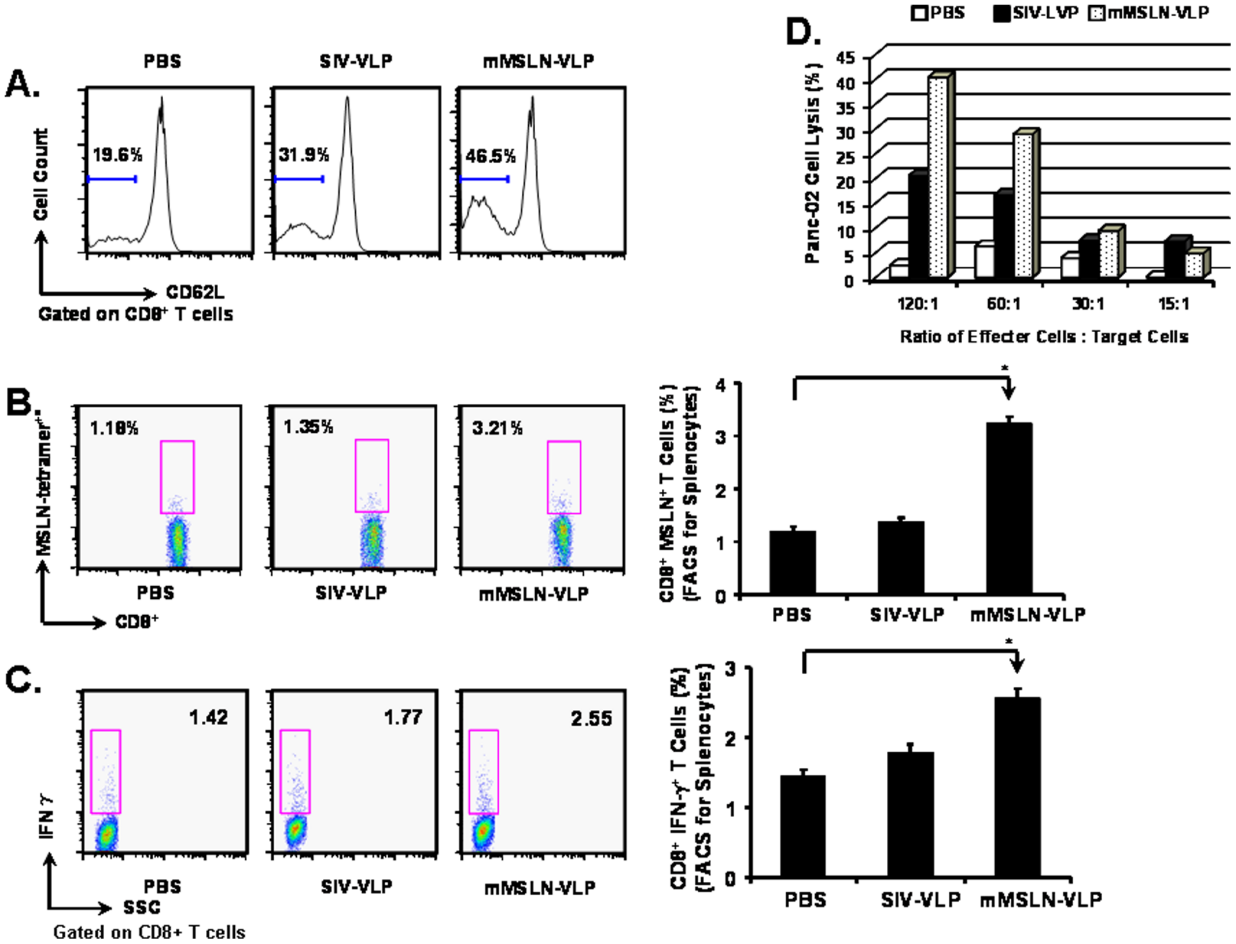
MSLN-specific functional CD8^+^ T cells in the mMSLN-VLPs immunized mice group. At 17^th^ day after tumor implantation and after one time VLP vaccination at day 3, mice were sacrificed for immune response analysis. FACSCalibur was used to acquire and FlowJo software was used to analyze the data. **A).** General CD8^+^ T cell activation in the PBS control group and the VLPs immunized mice groups. Cells were gated on CD8^+^ T cells, T cell activation marker CD62L expression were shown in the histogram. **B).** MSLN-specific tetramer^+^ CD8^+^ T cells induction. Splenocytes were stained with PE labeled MSLN-specific tetramer. **C).** MSLN-specific IFN-γ producing CD8^+^ T cell induction. Splenocytes were re-stimulated with MSLN peptide for 6 h in the presence of Golgi blockage compound *in vitro*. After fixation and permeabilization, the pre-treated splenocytes were stained by PE labeled anti IFN-γ antibody. **D).** MSLN-specific CTL induction against Panc02 cells. The splenocytes were re-stimulated *in vitro* for 6 days in the presence of IL-2. The pre-treated splenocytes were counted and used as effecter cells. Panc 02 cells were used as target cells. Effecter cells and target cells were mixed with various E:T ratio and incubated for 4 h. The percentage of lysis was calculated as: % Cytotoxicity = [(Experimental - Effecter Spontaneous - Target Spontaneous)/(Target Maximum - Target Spontaneous)] × 100. Shown were representative data selected from two independent experiments with five mice per group. (Statistically significances are shown, * indicates *p*<0.05.).

Our previous reports have shown that VLPs have a high capacity to activate Th1-oriented cellular immune responses and generate specific cytotoxic T lymphocytes in vaccinated mice [26], [27]. As shown in Fig. 2A, there was an increase in the frequency of CD8^+^CD62L^−^ T cells after mMSLN-VLP treatment (46.5% vs. 19.6% in PBS), suggesting that mMSLN-VLP can stimulate general CD8^+^ T cell activation.

To assess the generation of mMSLN-specific CD8^+^ T cells in murine splenocytes after immunization, an MHC-I: MSLN peptide tetramer was used to determine the percentage of mMSLN-specific CD8^+^ T cells. As shown in Fig. 2B, the percentage of tetramer+CD8^+^ T cells in mMSLN-VLP immunized groups was approximately 3-folds higher than that in the PBS control group (3.21% versus 1.18%). SIV-VLP immunization did not notably increase the frequency of mMSLN-specific CD8^+^ T cells (1.35%). Therefore, mMSLN-VLP immunization can induce mMSLN-specific CD8^+^ T cells.

Activated effector CD8^+^ T cells can be divided into two different groups: cytokine secreting CD8^+^ T cells and cytotoxic T lymphocytes [28]. After MSLN-specific peptide stimulation, MSLN-specific CD8^+^ T cells that secrete IFN-γ were detected by intracellular cytokine staining. As depicted in Fig. 2C, the percentage of MSLN-specific IFN-γ secreting effecter CD8^+^ T cells in the mMSLN-VLP immunized group was approximately 2-folds higher than in the PBS group (2.55% versus 1.42%). However, there was no enhancement in the frequency of these cells following SIV-VLP immunization (1.77%). To assess the capacity of VLP-induced CTLs to directly kill tumor cells, an *in vitro* tumor killing assay was performed using Panc02 cells as target cells which was shown to overexpress MSLN [21]. As shown in Fig. 2D, the cytolytic efficiency of CTLs to Panc02 cells was also shown to be the highest in the mMSLN-VLP vaccinated groups at a ratio of 120∶1. These results suggest that the specific effector CD8^+^ T cells induced by mMSLN-VLP immunization may play an important role in directly controlling tumor regression.

### mMSLN-VLP Immunization Suppresses Both Systemic and Tumor Infiltrating CD3e^+^CD4^+^foxp3^+^ Tregs

**Figure 3:**
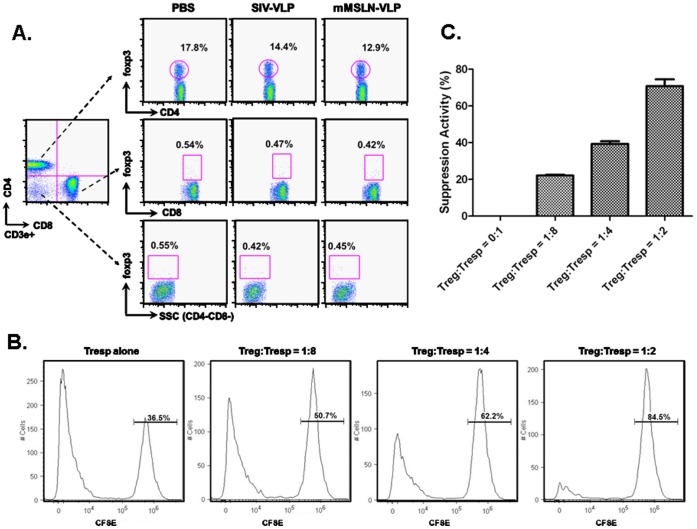
Characterization of foxp3^+^ Treg in tumor-bearing mice after VLP vaccination. A). At 12^th^ day after tumor implantation and after one time VLP vaccination at day 3, mice were sacrificed for immune response analysis. About 10^6^ splenocytes were incubated with the appropriately diluted fluorescent-conjugated antibodies including CD3e, CD4, CD8, and foxp3. Foxp3^+^ cells were analyzed in three T cell subsets: CD4^+^CD8^−^, CD4^−^CD8^+^, and CD4^−^CD8^−^. These were representative data from three independent experiments with at least three mice per group. B). CD4^+^Foxp3^+^ T cells isolated from tumor-bearing splenocytes suppressed Tresp cells proliferation. Co-culturing CD4^+^CD25^+^ Tregs and CD4^+^CD25^−^ Tresps (CFSE stained) at different ratios 1∶8, 1∶4, and 1∶2. CFSE labeled Tresps proliferation was analyzed by FACS. Numbers on the CFSE_high_ peak indicate percent of undivided cells. C). The suppressive activity of CD4^+^CD25^+^ Tregs on CD4^+^CD25^−^ Tresp cell proliferation at different Treg:Tresps ratios (x-axis). Y-axis indicates percent of suppression on Tresp cells proliferation activity.

Although we have shown that VLP immunization can reduce the number of CD4^+^foxp3^+^ Tregs cells in the murine spleen and in tumor tissue, the phenotype and biology of these cells still remainslargely unknown [21]. As shown in Fig. 3, almost all of the foxp3^+^ cells in tumor-bearing mice were present in the CD3e^+^CD4^+^ T cell population since CD3e^+^CD8a^+^foxp3^+^and CD3e^+^CD4^−^CD8a^−^foxp3^+^subsets were below 0.5%. The percentage of CD3e^+^CD4^+^foxp3^+^ Tregs following mMSLN-VLP immunization (12.9±1.83%) was lower than in the PBS control group (17.81±1.26%) (p<0.05). SIV-VLP immunization also led to a decrease in the population of Tregs, albeit, to a lower extent (14.4±2.19%) (p>0.05). Therefore, the decreased foxp3^+^ cells in tumor-bearing mice belonged to the conventional CD3e^+^CD4^+^foxp3^+^ Treg cell group, since VLPs did not induce or affect CD4^−^foxp3^+^ Treg cells in the murine spleen. Therefore, VLP immunization can reduce the systemic population of CD3e^+^CD4^+^foxp3^+^ Tregs in tumor-bearing mice.

To demonstrate that CD4^+^foxp3^+^ T cells isolated from splenocytes of tumor-bearing mice possess suppressive activity, we performed a CFSE-based Treg suppression assay. To obtain live cells for this 5 day co-culture assay, we used CD4 and CD25 surface markers to isolate CD4^+^CD25^+^ Tregs. We performed intracellular staining of the Foxp3 marker to confirm that the majority of isolated CD4^+^CD25^+^ Tregs were also CD4^+^foxp3^+^ T cells. As demonstrated in Fig. S1, gated on CD4^+^CD25^+^ cells, about 87% of the cells stained positive for Foxp3^+^. The CFSE-based Treg suppression assay was performed by co-culturing CD4^+^CD25^+^ Tregs and CD4^+^CD25^−^ responder T cells (Tresps) (CFSE stained) isolated from the spleens of PC tumor-bearing mice. As shown in Fig. 3B, when no Treg cells were added, most of the Tresp cells underwent proliferation and only 36.5% remained undivided. As the ratio of Treg cells to Tresp cells increased in the co-culture, suppression of Tresp cell proliferation was observed. The percentage of undivided Tresp cells increased from 36.5% (no Treg added) to 50.7%, 62.2%, and 84.5% at Treg:Tresp ratios of 1∶8, 1∶4, and 1∶2, respectively. As depicted in Fig. 3C, the suppressive activity of CD4^+^CD25^+^ Tregs on CD4^+^CD25^−^ Tresp cell proliferation was 22% when 12.5% Tregs were present in the co-culture, and it increased to 40% and 76% when 25% and 50% of Tregs were present, respectively. These data strongly supports the notion that CD4^+^foxp3^+^ T cells in tumor-bearing splenocytes possess suppressive activity.

To determine the phenotype of foxp3^+^ Tregs in tumor tissues, immunofluorescence staining was performed. In tumor tissues from mice that did not receive mMSLN-VLP immunization, we found that most foxp3^+^ Tregs were infiltrated in the tumor tissue from the blood vessels and located near the tumor vasculature (Fig. S2A). Additionally foxp3^+^ Tregs in the tumor tissue were of the phenotype CD4^+^CD8a^−^ (Fig. S2B), and a large portion of CD8a^+^ T cells (green stained cells) were located next to foxp3^+^ T cells (red stained cells) (Fig. S2C). Comparing the number of foxp3^+^ cell numbers between immunized and non-immunized groups, foxp3^+^ cells are reduced in VLP-immunized mouse tumor tissues (Fig. S2D). This observation implies that foxp3^+^ Tregs may rely on direct interaction with CD8a^+^ T cells to exert its suppressive effect on CD8^+^ T cell anti-tumor function in the non-immunized mouse group, whereas, VLP immunization reduces the foxp3^+^ Treg population and thus the suppression exerted by foxp3^+^ Tregs in the tumor tissue may be alleviated.

### High Levels of IL-6 induction by mMSLN-VLP is Partially Responsible for the Treg Down-regulation

**Figure 4:**
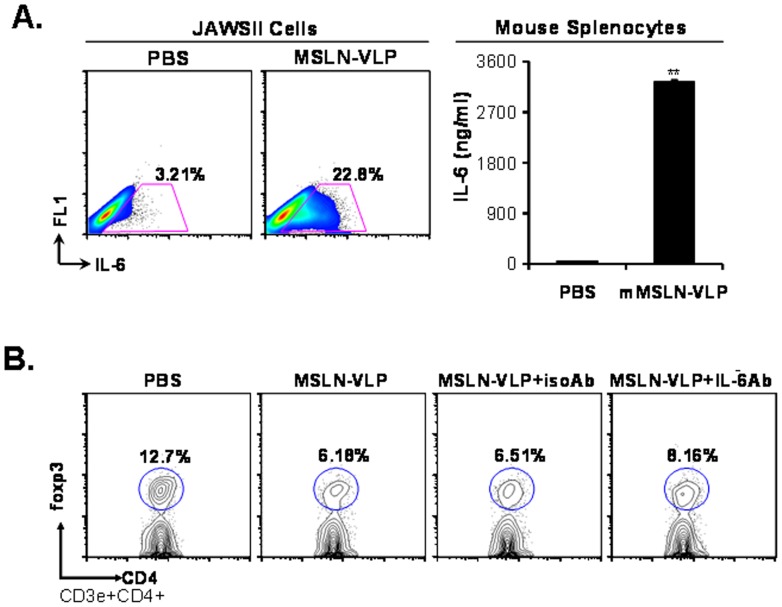
Enhanced IL-6 production in DCs and lymphocytes upon mMSLN-VLP treatment *in vitro* and IL-6 is partially responsible for foxp3^+^ Treg cells reduction. **A).** Increased IL-6 producing JAWSII cells and elevated IL-6 levels in lymphocytes culture media upon mMSLN-VLP treatment. JAWSII cells were stimulated with MSLN-VLP (5 µg/ml) for 12 h and intracellular IL-6 staining was performed. Lymphocytes were prepared from spleen of naïve B6 mice. After incubation with MSLN-VLP for 24 h, supernatant was collected to determine IL-6 concentrations by Bio-Plex. **B)**. Partial rescue of suppressive effect of mMSLN-VLP stimulation on foxp3^+^ Treg cells by IL-6 neutralization. MSLN-VLP with or without IL-6 neutralizing Ab or isotype control Ab were added to lymphocytes culture for 3 days. The foxp3^+^ cells were stained by using foxp3 intracellular staining kit. Data was acquired for flow cytometry analysis gated on CD3^+^ and CD4^+^ T cells. Shown were representative data from three to five independent experiments. (Statistically significances are shown, ** indicate *p*<0.01.).

IL-6 was reported to inhibit natural occurring foxp3^+^ Tregs. To assess whether mMSLN-VLP immunization could induce IL-6 production, both splenocytes and JAWSII cells were used for stimulation *in vitro* with mMSLN-VLPs. As shown in Fig. 4A, there was a notable increase in IL-6 secreting DCs in the mMSLN-VLP treated group compared with the PBS control group (22.8% versus 3.21%). In addition, enhanced IL-6 secretion from murine splenocytes was also detected upon the mMSLN-VLP stimulation. There was 100-fold increase in the levels of IL-6 secreted from splenocytes in the mMSLN-VLP treated group when compared with the PBS control group. This suggests that mMSLN-VLP has the capacity to stimulate lymphocytes, specifically DCs, and induce their secretion of high levels of IL-6.

To address whether the reduction in foxp3^+^ Tregs by mMSLN-VLP treatment was mediated by IL-6, IL-6 neutralizing antibody was applied during VLP stimulation and an isotype control antibody was used as a control. As shown in Fig. 4B, the percentage of foxp3^+^ Tregs was 12.7% in CD3^+^CD4^+^ gated cells from fresh naïve B6 mice after 3 days of incubation. However, the percentage of foxp3^+^ Tregs was reduced by mMSLN-VLP treatment with or without isotype control antibody (6.51% and 6.18%). Consequently, IL-6 neutralizing antibody was able to partially rescue the reduction in the frequency of foxp3^+^ Treg cells by approximately 32% (8.16% compared with 6.18% in mMSLN-VLP only treatment). These data indicate that IL-6 may play a role in the reduction of foxp3^+^ Tregs by mMSLN-VLP immunization.

### mMSLN-VLP Immunization can Selectively Suppress a Subset of CD4^+^ Foxp3^+^ICOS^−^ Tregs

**Figure 5:**
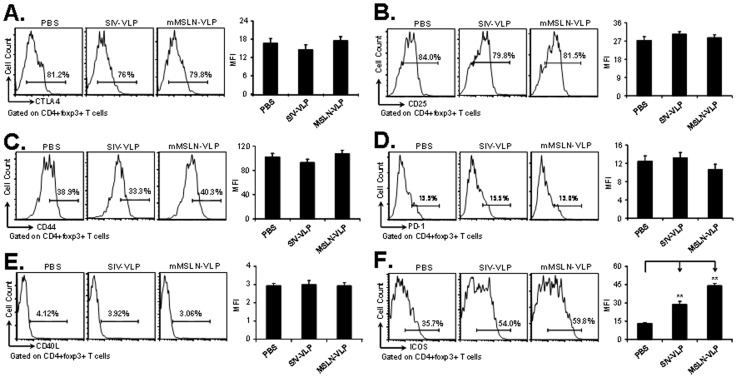
Functional proteins expressing on foxp3^+^ Treg in tumor-bearing mice spleen after VLP vaccination. At 12^th^ day after tumor implantation and after one time VLP vaccination at day 3, mice were sacrificed for immune response analysis. About 10^6^ splenocytes were incubated with the appropriately diluted fluorescent-conjugated antibodies including CD4, foxp3, CTLA-4, CD25, CD44, PD-1, CD40L, and ICOS. FACSCalibur was used to acquire and FlowJo software was used to analyze the data. Cells were gated on CD4^+^foxp3^+^ population and histogram of different molecule expression in different experimental groups were presented in **A).** CTLA-4; **B).** CD25; **C).** CD44; **D).** PD-1; **E).** CD40L; **F).** ICOS. Shown were representative data from three experiments with at least three mice per group. (Statistically significances are shown, ** indicate *p*<0.01.).

Functional molecules such as CTLA-4, CD25 and CD44 expressed on the surface of foxp3^+^ Tregs are essential in dictating the homing, function and survival capacity of these cells. To further address how VLPs affect foxp3^+^ Treg biology, several important functional molecules were stained. Compared with the PBS group, there were no obvious differences in the frequencies of CD4^+^foxp3^+^CTLA-4^+^ Tregs (Fig. 5A), CD4^+^foxp3^+^CD25^+^ Tregs (Fig. 5B), CD4^+^foxp3^+^CD44^hi^ Tregs (Fig. 5C) following VLP immunization. The inhibitory molecule PD-1 and co-stimulatory molecule CD40L had a low expression level in foxp3^+^ Tregs (Fig. 5D). The percentage and MFIs for CD4^+^foxp3^+^PD-1^+^ and CD4^+^foxp3^+^CD40L^+^ Tregs in the VLP groups were very similar to those values in the PBS control group. However, compared with PBS treatments (35.7%), the percent of CD4^+^foxp3^+^ICOS^+^ Tregs in VLP treated groups was increased. The highest percentage of CD4^+^foxp3^+^ICOS^+^ Treg subset was observed after mMSLN-VLP immunization (59.8%). The percentage in the SIV-VLP treated mice was 54.0% (Fig. 5F). By correlating the above data to the decrease in absolute number of foxp3^+^ cells and the increase in the percentage of ICOS^+^ cells in the foxp3^+^ Treg group, these data suggests that the reduction in foxp3^+^ Tregs corresponds to the population of CD4^+^foxp3^+^ICOS^−^ Tregs but not to the CD4^+^foxp3^+^ICOS^+^ Tregs. ICOS may therefore play a critical role in the tumor suppressive function exerted by Tregs in tumor-bearing mice. These results suggest that even though mMSLN-VLP immunization can reduce the overall frequency of Treg cells these are mainly from the CD4^+^ foxp3^+^ICOS^−^ Treg cell subset, but those Tregs belonging to the CD4^+^foxp3^+^ICOS^+^ subset appear to be maintained.

To determine the presence of both Treg cell subsets in pancreatic cancer patients, tissue samples were analyzed by immunofluorescence staining. As shown in Fig. S3A, there was a high density of CD3^+^foxp3^+^ Tregs in pancreatic tumor tissues and more than half of those foxp3^+^ Tregs were ICOS^+^ (Fig. S3B). This data suggests that foxp3^+^ Tregs play an important role in the pathogenesis of pancreatic cancer, and both foxp3^+^ICOS^+^ and foxp3^+^ICOS^−^ Tregs subpopulations are involved in immune suppression in tumor tissues.

### mMSLN-VLP Treatment Activates pDCs and Upregulates ICOSL on pDCs through IL-6 to Maintain CD4^+^foxp3^+^ICOS^+^ Treg Cells but not CD4^+^foxp3^+^ICOS^−^ Treg Cells

**Figure 6:**
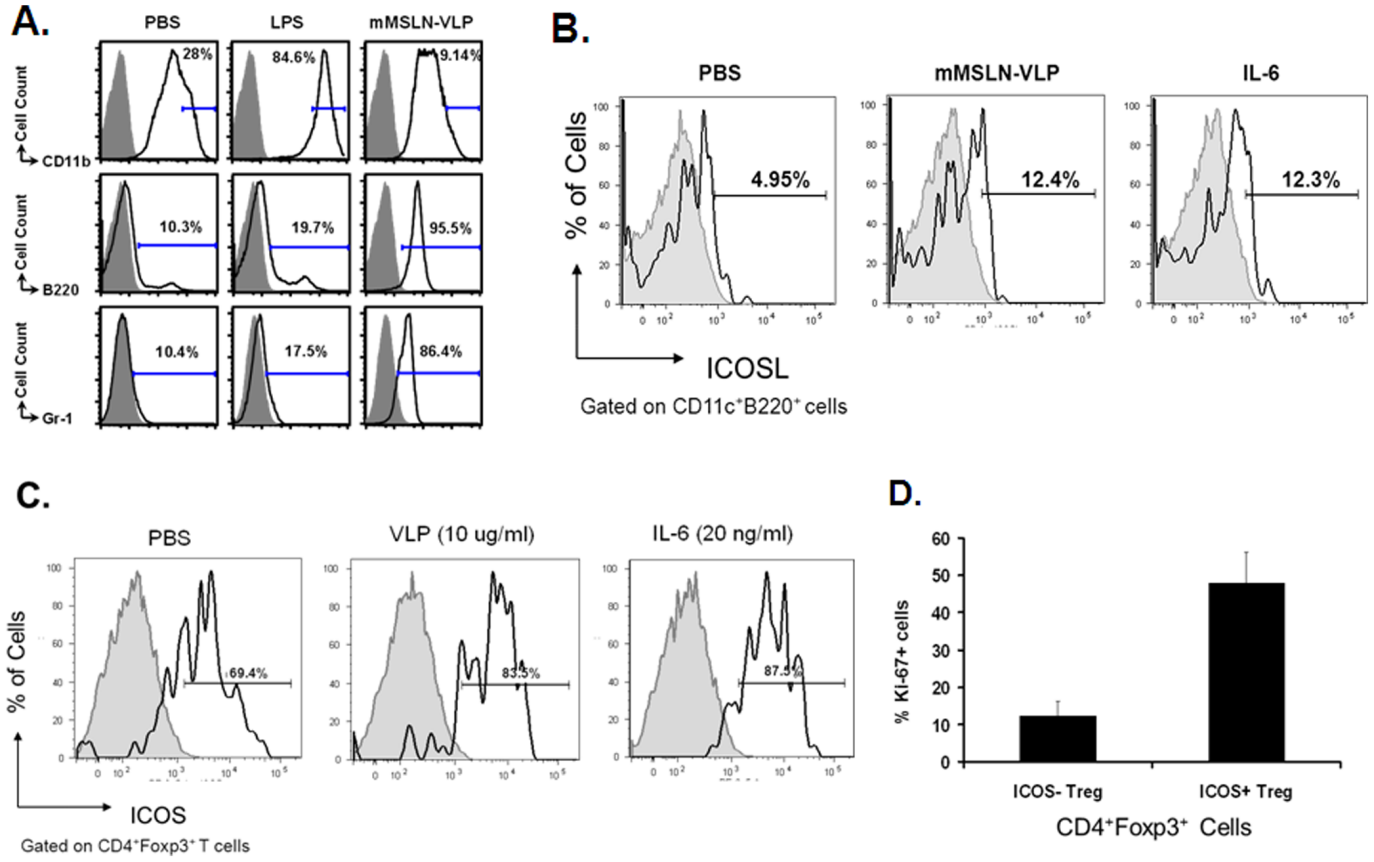
Activation of pDCs-like cells and reduction of CD4^+^foxp3^+^ICOS^−^ Treg upon mMSLN-VLP stimulation. About 10^7^ JAWSII cells were stimulated with either PBS, LPS (1 µg/ml), or MSLN-VLP (5 µg/ml) in complete alpha media with 5 ng/ml rmGM-CSF. After 24 h incubation, cell surface markers were stained and acquired by FACSCalibur. **A).** Expression of CD11b, B220, and Gr-1 upon different treatment of PBS, LPS, or mMSLN-VLP in JAWSII cells. **B).** Expression of ICOSL in PBS, mMSLN-VLP or IL-6 induced pDCs. **C).** Expression of ICOS on CD4^+^foxp3^+^ Tregs. **D)**. Percentage of Ki-67 staining cells in both CD4^+^foxp3^+^ICOS^+^ Tregs and CD4^+^foxp3^+^ICOS^−^ Tregs. Multiple cell surface markers were stains and analysis of subpopulation phenotypes was done by using FACSCalibur and FlowJo software. Shown were representative data from three to six independent experiments. (Statistically significances are shown, ** indicate *p*<0.01.).

Previous reports have shown that pDCs express high level of ICOSL, and the interaction between ICOS and ICOSL is important for the homeostasis of CD4^+^foxp3^+^ICOS^+^ Treg cells [13], [29]. Since mMSLN-VLP immunization affects the frequency of CD4^+^foxp3^+^ICOS^−^ Treg cells but not CD4^+^foxp3^+^ICOS^+^ Treg cells, one of the possible explanations for this preferential maintenance of CD4^+^foxp3^+^ICOS^+^ Treg cells could be due to mMSLN-VLP mediated upregulation of ICOSL on pDCs. To study whether mMSLN-VLP could activate pDCs, a mouse DC cell line, JAWSII, was used. LPS was used as a control for stimulating DCs. As shown in Fig. 6A, LPS stimulation significantly increased CD11b expression compared with PBS treatment (84.6% versus 28.0%). Nevertheless, mMSLN-VLP largely decreased the expression of CD11b (9.14%). In contrast, the expression of B220 and Gr-1 was dramatically up-regulated following mMSLN-VLP treatment (95.5% and 86.4%). B220 and Gr-1 have been identified as primary markers of pDCs. There were only low levels of B220 and Gr-1 expression following PBS and LPS treatments (10.3% and 10.4% in PBS, 19.7% and 17.5% in LPS treatment). These data indicates that mMSLN-VLPs can promote pDCs-like cells while LPS has a preference for activating cDC-like cells.

To determine whether mMSLN-VLP activated pDCs-like cells express elevated ICOSL, or whether mMSLN-VLP induced IL-6 can upregulate ICOSL on pDCs, PBS, mMSLN-VLP (10 µg/ml) or IL-6 (20 ng/ml) were used to stimulate splenocytes and stain for surface ICOSL on gated pDCs. As shown in Fig. 6B, the percentage of ICOSL-expressing cells in mMSLN-VLP activated pDCs or IL-6 activated pDCs were 12.4% and 12.3%, respectively, which were significantly increased compared with PBS activated DCs (4.95%). These data suggest that mMSLN-VLP or IL-6 activated pDCs-like cells do express high levels of ICOSL.

We have shown that mMSLN-VLP vaccination induced high levels of IL-6 production which is partially responsible for the reduction in Treg cell frequency, but the effect IL-6 has in the CD4^+^foxp3^+^ICOS^+^ Tregs subpopulation still needs to be determined. We therefore assessed whether mMSLN-VLP or IL-6 could also reduce total CD4^+^foxp3^+^ Tregs while maintaining a high percentage of CD4^+^foxp3^+^ICOS^+^ Tregs *in vitro*. Mouse splenocytes were stimulated with either PBS, mMSLN-VLP (10 µg/ml), or IL-6 (20 ng/ml). While we did observe significant CD4^+^foxp3^+^ Treg downregulation in both the mMSLN-VLP and IL-6 treatment groups, interestingly, we observed that IL-6 had a similar effect as mMSLN-VLP treatment which also led to an increase in the frequency of CD4^+^foxp3^+^ICOS^+^ Tregs cells (Fig. 6C). To further examine the property of this subpopulation of Treg cells and to determine whether ICOS expression is related to the proliferation of the CD4^+^foxp3^+^ICOS^+^ Treg subpopulation, Ki-67 expression was used to determine the cycling of the cells. As shown in Fig. 6D, we found a significant proportion of Ki-67 positive cells within the ICOS^+^ Tregs vs. ICOS- Tregs (47.8±8.4% vs. 12.3±3.8%, p<0.001). This indicates that even though mMSLN-VLP induced IL-6 reduces the overall frequency of Treg cells it may also be responsible for the increased proportion of remaining CD4^+^foxp3^+^ICOS^+^ Treg subset through enhanced proliferation of ICOS^+^ cells presenting an double edged sword.

## Discussion

The rapid development of cancer vaccines provides great promise for tumor prevention and therapeutics [4]. Unlike other tumor therapies, vaccines have many advantages such as the development of a memory response, specific targeting of cancerous cells and fewer side-effects among others. In this study, we demonstrated that mMSLN-VLP immunization could break the tolerance to self mMSLN and mount a strong immune response against mMSLN, and therefore, effectively reduce tumor size and notably prolong the survival of tumor-bearing mice in an orthotopic pancreatic cancer model. MSLN-specific CD8^+^ T cells can be effectively induced by mMSLN-VLP treatment and have been found to contribute to the suppression of tumor growth. Furthermore, the number of foxp3^+^ Treg cells was dramatically decreased in both the murine spleen and tumor tissues and specifically a subset of CD4^+^foxp3^+^ICOS^−^ Tregs was reduced upon VLP immunization. It was further found that mMSLN-VLP can activate pDC-like cells which secrete elevated levels of IL-6 and enhance ICOSL expression, which support the survival of CD4^+^foxp3^+^ICOS^+^ Treg cells but not CD4^+^foxp3^+^ICOS^−^ Treg.

Cytotoxic T lymphocytes play a central role in adaptive anti-tumor immunity by directly removing cancer cells that express peptide bound MHC-I. Here, we used MSLN-specific tetramer staining to show the generation of MSLN-specific CD8^+^ T cells by mMSLN-VLP immunization. In addition, MSLN-specific intracellular cytokine staining and MSLN-specific CTL assays further confirmed that mMSLN-VLP immunization has the ability to induce a MSLN-specific CD8^+^ T cell response which is beneficial for suppressing tumor growth. In general, CD8^+^ T cells were activated likely by various immune epitopes present in the VLPs and redistributed in the absence of the homing molecule CD62L after vaccination. The specificity of the immune response generated was confirmed by the population of CD8^+^ T cells recognizing the immune MSLN epitope GQKMNAQAI as shown by positive tetramer staining. The strong ability of mMSLN-VLP to induce an efficient CD8^+^ T cell response in Panc02-bearing mice was further elucidated in this study. Other vaccine strategies such as DNA vaccines [30], [31], DC vaccines [32], tumor cell/extracts [33] showed the capacity to generate specific effecter CD8^+^ T cells, but there is no direct comparison of which vaccine has a higher ability to induce a CD8^+^ T cell response. It was reported, however, that vaccination with VLP was superior to that of plasmid DNA in tumor protection [34].

Many tumor models have been used to show the role of Tregs in promoting tumor growth by exerting their suppressive effect on other immune cells. Tregs have been found to directly inhibit not only CD4^+^ helper T cells and CD8^+^ cytotoxic T cells but also NK cells following cancer immunotherapy [35]. Vaccination against foxp3 through transfection of DCs showed an enhancement of anti-tumor immunity [36]. Consistent with the results obtained with other inactive virus particles used as tumor vaccines [37], we also showed that mMSLN-VLP immunization can reduce the frequency of Tregs which alleviated their suppressive effect on CD8+ T cells and thus reduce the tumor burden in mice. In addition, we found that reduction of foxp3^+^ Tregs was partially mediated by increased levels of IL-6 which were secreted by VLP-activated pDCs-like cells. The detailed mechanism of Treg-mediated suppression remains unclear. Different mechanisms of contact-mediated suppression have been reported including granzyme-dependent, CTLA-4-dependent, and TGF-β dependent suppression [38]–[40]. We found that there is a possible direct interaction between Treg and CD8+ T cells in the tumor site which indicates the suppressive effect may be exerted directly by Tregs onto CD8+ T cells. Robertson et al [41] has also shown that the Friend virus-induced Treg suppression effect was mediated by direct T cell-T cell interactions and occurred in the absence of APCs.

Among many vital molecules on foxp3^+^ Tregs, such as CD25, CTLA-4, CD44, ICOS and PD-1, only ICOS expression was dramatically altered by VLP treatment. CD4^+^foxp3^+^ICOS^+^ and CD4^+^foxp3^+^ICOS^−^ Tregs have been shown to have distinct life cycles and functions [13]. CD4^+^foxp3^+^ICOS^+^ Tregs are induced by DCs through ICOSL-ICOS interaction. They secrete IL-10 to suppress DC function and TGF-β to suppress T cell function. On the other hand, CD4^+^foxp3^+^ICOS^−^ Treg cells are induced by DC through CD80/CD86-CD28. This subset produces TGF-β only. Similar to other findings [14], our results showed that in both tumor and spleen, the majority of the Treg population was CD4^+^foxp3^+^ICOS^+^ (approximately 65%) in the Panc02 tumor model, suggesting that CD4^+^foxp3^+^ICOS^+^ Tregs plays a critical suppressive role to the immune system that benefits tumor growth. As an analog of virus, VLP can widely promote DC maturation [42], and our data further showed that VLP stimulated DC activation and increased pDCs-like cells with up-regulation of B220, Gr-1 and ICOSL but not CD11b *in vitro*, subsequently supporting CD4^+^foxp3^+^ICOS^+^ Treg survival. Our data also shows that mMSLN-VLP stimulated pDCs secrete higher levels of IL-6. This cytokine in turn can stimulate ICOSL expression on pDCs which supports the survival of ICOS+ Tregs subpopulation, and ICOS^+^ Treg cells have a higher proliferation capacity compared to ICOS- Treg cells. The direct effect of IL-6 was shown in figure 6. This data further shows the important role of IL-6 and IL-6 induced high ICOSL expression on pDCs which collectively acts on the maintenance of ICOS^+^ Tregs. Our results support the findings by Kornete et al. where they showed that ICOS-ICOSL selectively promotes Foxp3 Treg functions. The ICOS^+^ Treg subset, in contrast to the ICOS^−^ Treg subset, has prominent proliferative capacity, and therefore, ICOS-ICOSL interaction is critical for the homeostasis of Tregs. This also explains that while VLP immunization can reduce total number of Tregs, it mostly only inhibits the CD4^+^foxp3^+^ICOS^−^ Treg subset. Although it is well-established that only a number of Treg cells is vital for tightly controlling immunity in both humans and mice, due to no antigen specificity to targeted cells [43], we believe additional strategies will be needed to help reduce the CD4^+^foxp3^+^ICOS^+^ Treg subpopulation in order to completely alleviate the immune suppression effect exerted by residual Tregs upon VLP treatment. Once again, our data indicates the critical role of ICOS in the maintenance of foxp3^+^ Treg cells in the tumor microenvironment. This mechanism has been indirectly supported by previous studies, which demonstrated that ICOS may have a key role in maintenance and function of Treg cells [29], [44].

In summary, we have shown effective control of mouse pancreatic tumor growth and further demonstrated the possible mechanism of anti-tumor immunity mediated by mMSLN-VLP immunization. However, even though the high levels of IL-6 produced by mMSLN-VLP activated pDC-like cells was at least partly responsible for reducing the frequency of Treg cells, it also increased the expression of ICOSL on pDC-like cellswhich supported the survival of CD4^+^foxp3^+^ICOS^+^ Tregs. Therefore, in addition to mMSLN-VLP immunization, which can reduce the frequency of CD4^+^foxp3^+^ICOS^−^ Treg cells, combination strategies are needed to further affect the CD4^+^foxp3^+^ICOS^+^ Treg subpopulation.

## Materials and Methods

### Antibodies, Reagents, and Cells

Rabbit anti-MSLN polyclonal antibody was generated from Genemed, and anti-MSLN monoclonal antibody was purchased from Abcam. Other Abs were purchased from eBiosciences, BD Biosciences and BioLegend, which includes FITC conjugated Abs against CD4 (GK1.5), CD8a (53-6.7), CD44 (IM7), ICOS (C398.4A) and PD-1 (J43); PE conjugated Abs against CD3e (145-2C11), CD4 (H129.19), CD8a (53-6.7), CD152 (UC10-4B9), CD40L (MR1), B220 (RA3-6B2) and IFNγ (XMG1.2); PerCP conjugated Abs against B220 (RA3-6B2), CD4 (RM4-5) and CD8a (53-6.7); and APC conjugated Abs against CD8a (CT-C8a), CD62L (MEL-14) and B220 (RA3-6B2). Mouse IL-6 recombinant protein was purchased from eBioscience Inc. (San Diego, CA). PE or APC labeled anti-mouse/rat FoxP3 staining set (FJK-16 s), IL-6 neutralizing Ab (MP5-20F3), and Rat IgG1 Ab isotype control Ab. JAWSII, a mouse DC cell line, was obtained from ATCC and cultured in alpha minimum essential medium with 5 ng/ml murine GM-CSF (eBioscience Inc, San Diego, CA) and 20% FBS.

### VLP Production and Characterization

The production and purification of VLPs were previously described [45]. Briefly, Sf9 insect cells were co-infected with recombinant baculovirus (rBV) expressing SIVmac239 gag and with rBV expressing murine MSLN for 3 days, and then the cells were collected and centrifuged at 2,500 RPM for 20 min (GPR desktop centrifuge; Beckman Coulter, Fullerton, CA). The VLP-containing supernatant was then filtered through a 0.45-µm filter. VLPs were then pelleted at 300,000×g for 1 h at 4°C and re-suspended in PBS followed by purification with a 20∼60% continuous sucrose gradient at 100,000×g for 16 h at 4°C. The VLP-containing band was then carefully collected and dialyzed against cold PBS with a 10,000 m.w. cut-off membrane overnight at 4°C. The VLPs were then pelleted again at 300,000×g for 1 h at 4°C and re-suspended in PBS overnight. Western blot with anti-mouse MSLN antibodies were performed to determine MSLN protein incorporation onto the VLPs. The total protein concentration of the VLP prep was measured using a Bio-Rad protein assay kit. The endotoxin level was quantitated using the Limulus amebocyte assay kit (Associates of Cape Cod, Woods Hole, MA) and controlled under 0.0041 EU/µg.

### Orthotopic Pancreatic Cancer Mouse Model and Vaccination

The murine pancreatic cancer cell line, Panc02 (syngeneic to C57BL/6 mice (H-2^b^)), was cultured as originally described [21]. The orthotopic pancreatic cancer mouse model was previously described [21]. Tumor cells were implanted at day 0. Three immunizations at days 3, 13, and 21 were applied by i.p. route. Three groups: PBS (100 µl), SIV-VLP (100 µg/100 µl), mMSLN-VLP (100 µg/100 µl). The production and purification of VLPs were previously described ^20^, [45]. For immune response studies, at day 17 after tumor implantation, mice (5 mice per group) were sacrificed, tumors were removed for the size measurement, and splenocytes were collected for immune response analysis. For the survival study, survival of each mouse (10 mice per group) was recorded. Animals were euthanized when they became moribund during the observation period, and the time of euthanization was recorded as the time of mortality.

### Interferon γ, IL-6, and foxp3 Intracellular Staining

Intracellular staining of cytokines was performed using the manufacturer’s protocol (BD Biosciences, San Jose, CA). Single cell suspensions were diluted in DMEM supplemented with 10% FBS. Approximately, 3×10^7^ cells were added to each well of a 6-well plate followed by the addition of 100 µl of stimulation mixture (final concentrations: 10 µg/ml peptide, 10 U/ml murine IL-2 and 2 µM monesin or 5 µg/ml brefeldin A). Cells were re-stimulated at 37°C for more than 6 h (monesin or brefeldin A was added at 2 h after stimulation), washed three times, and adjusted to 2×10^7^ cells/ml. Cells (50 µl) were then added to each well of a micro-titer plate followed by the addition of 50 µl of an antibody mixture at the appropriate dilutions. Cells were incubated on ice for 30 min, washed three times and re-suspended in 200 µl of BD Cytofix/CytoPerm solution. Cells were then incubated on ice for 20 min followed by two washes with 200 µl of BD Perm/Wash buffer. The cells were then re-suspended in 100 µl of Fc blocking solutions. After blocking for 15 min, 50 µl of cytokine staining master buffer was added. After 30 min incubation on ice, the cells were washed three times with BD Perm/Wash and re-suspended in 300 µl of FACS buffer for acquisition on a FACSCalibur (Becton Dickinson, San Jose, CA).

Intracellular staining of foxp3 was performed by following the manufacturer’s protocol (eBioscience, Inc., San Diego, CA). In brief, following surface staining and subsequent washes, cells were re-suspended by pulse vortexing and 250 µl of freshly prepared Fixation/Permeabilization working solution was added. After incubation overnight at 4°C in the dark, 200 µl of permeabilization buffer was added to wash the cells three times by using the centrifugation and decanting procedure. Titrated anti-mouse/rat Foxp3 (FJK-16 s) antibody in permeabilization buffer was then added and incubated on ice for 30 min in the dark. After washing the cells three times with 200 µl of permeabilization buffer, the cells were re-suspended in 300 µl of PBS and run in a FACSCalibur.

### Cytotoxic T lymphocyte Assay

The generation of antigen specific CTLs in vaccinated tumor-bearing mice was measured by using the CytoTox 96 Non-Radioactive Cytotoxicity Assay as shown previously [21]. Briefly, splenocytes were stimulated with MSLN-specific peptide (GQKMNAQAI) at a concentration of 2 µM for 6 days in the presence of 10 U/ml of murine IL-2. These cells were then used as effecter cells. Panc02 cells were used as target cells. EL4 was used as a negative control target cells. Different effecter-to-target cell ratios were used and the specific lysis of target cells was measured and calculated using the formula indicated in the CytoTox 96 assay kit. The percentage of specific lysis presented in the figure are the percent of specific lysis minus the non-specific killing negative control. The percent of specific lysis was presented by minus the non-specific killing negative control. MSLN-specific tetramer was made by the protein core at BCM.

### CFSE-based Treg Suppression Assay

CD4^+^CD25^+^ Tregs and CD4^+^CD25^−^ Tresp were isolated from the spleens of tumor-bearing mice with the EasySep mouse CD4^+^ T cell pre-enrichment and CD25 positive selection kits, used as according to the manufacturer’s instructions (Stem Cell Technologies, Vancouver, BC, Canada). The co-culture of Tregs and Tresp was performed as previously described [46]. Briefly, freshly isolated CD4^+^CD25^−^ Tresp were suspended at 2×10^6^ cells/ml and incubated with 5 µM carboxyfluorescein succinimidyl ester (CFSE) in PBS +0.1% BSA for 10 min at room temperature. The labeling reaction was quenched by adding ice-cold PBS, and cells were washed and resuspended at 5×10^5^ cells/ml in complete RPMI media (RPMI 1640+10% FBS). Tregs were suspended in complete RPMI at 2.5×10^5^ cells/ml and added to a 96 well round-bottom plate. Serial dilution of the Tregs was performed such that when CFSE-labeled Tresp were added to the Tregs, Treg:Tresp ratios of 1∶2, 1∶4 and 1∶8 could be achieved. Tresp cells alone were used as the negative control. The plate was incubated at 37°C, 5% CO_2_ for 5 days. On the 5^th^ day, cells were washed twice with flow cytometry buffer (DPBS +2% FBS +0.1% sodium azide), and resuspended in the same buffer for high-throughput analysis on a flow cytometer (Attune Cytometer, Applied Biosystems, Grand Island, NY), using an excitation laser of 488 nm and emission filter of 530/30 nm. The suppressive activity of Tregs towards Tresp was expressed as the relative inhibition of percentage of CFSE_low_ proliferating cells: [100× (1-%CFSE_low_ CD4^+^CD25^−^ Tresp in co-culture/%CFSE_low_ CD4^+^CD25^−^ Tresp alone)].

### VLP Stimulation *in vitro*

Various stimulators (final concentration: PBS, LPS at 1 µg/ml, mMSLN-VLP at 5 µg/ml) were added to 6 well (6 ml) flat bottom plates with 2×10^5^ splenocytes. The cells were then incubated for 4 days at 37°C. Cell surface markers were analyzed by FACS analysis as described previously [27], [42].

### Bio-Plex IL-6 Assay and IL-6 Blocking Assay

The concentration of IL-6 in cell supernatants was determined by using the Bio-Plex mouse IL-6 Assay kit (Bio-Rad Laboratories) according to manufacture’s suggestions. For IL-6 blocking assay, IL-6 neutralizing antibody (0.1 ng/ml) or isotype control antibody (0.1 ng/ml) was added to the cells that treated with mMSLN-VLP (final concentration 5 µg/ml).

### Statistical Methods

Quantitative results were shown as Mean ± SD. Statistical analysis was done by Student’s t test for paired data between control and treated groups or one-way ANOVA when using data from multiple groups. Kaplan-Meier survival test was done for comparing the survival curves between the vaccinated and control groups. *P*<0.05 was considered as a significant result.

## Supporting Information

Figure S1 Marker phenotypes of CD4^+^CD25^+^ Tregs. Cells were intracellularly stained with Foxp3 Abs and analyzed on gated CD4^+^CD25^+^ Tregs. Grey peak is the isotype control. The peak shifted to the right is Foxp3^+^ staining cells.
(PPT)

Figure S2 Characterization of Foxp3^+^ Treg in mice tumor tissues. Tumor tissues from PBS control group were used to characterize Foxp3^+^ cells in non-VLP immunized mice tumor tissues. Anti-Foxp3-PE antibody was used to stain Foxp3^+^ cells (red cells). Anti-CD3e-, anti-CD4-, and anti-CD8a- Abs conjugated with FITC were used to stain CD3^+^, CD4^+^, and CD8^+^ cells (green cells), respectively. **A).** CD3e^+^ T cell (green) and Foxp3^+^ cell (red) staining in tumor tissues; **B).** CD4^+^ or CD8^+^ T cell (green) and Foxp3^+^ cell (red) staining in tumor tissues. **C).** High power view of CD8^+^ T cells (green cells) and Foxp3^+^ T cells (red cells) localization in tumor tissues. **D).** At 12th day after tumor implantation and after one time VLP vaccination at day 3, mice were sacrificed for immune response analysis to compare the Foxp3^+^ T cell numbers in tumor tissues from three experimental groups. Results shown represents 5 different sample staining.
(PPT)

Figure S3 Characterization of Foxp3^+^ Treg in pancreatic cancer patient tumor tissues. Immunofluorescence staining was performed on frozen tissue blocks. **A).** CD3^+^Foxp3^+^ T cell staining in human PC tumor tissues. Anti-Foxp3-PE antibody was used to stain Foxp3^+^ cells (red cells). Anti-CD3e Ab conjugated with FITC was used to stain CD3 cells (green cells). **B).** Foxp3^+^ICOS^+^ T cell subpopulation staining in human tumor tissues. Anti-Foxp3-PE antibody was used to stain Foxp3^+^ cells (red cells). Anti-ICOS Ab conjugated with FITC was used to stain ICOS^+^ cells (green cells). Results shown represents 5 different sample staining.
(PPT)
