# Supplementary figures and images for: Mesothelin Virus-Like Particle Immunization Controls Pancreatic Cancer Growth through CD8+ T Cell Induction and Reduction in the Frequency of CD4+foxp3+ICOS− Regulatory T Cells
Source: PLoS One. 2013 Jul 9;8(7):e68303. doi: 10.1371/journal.pone.0068303 (PMC3706370; doi:10.1371/journal.pone.0068303)

## Slide 1
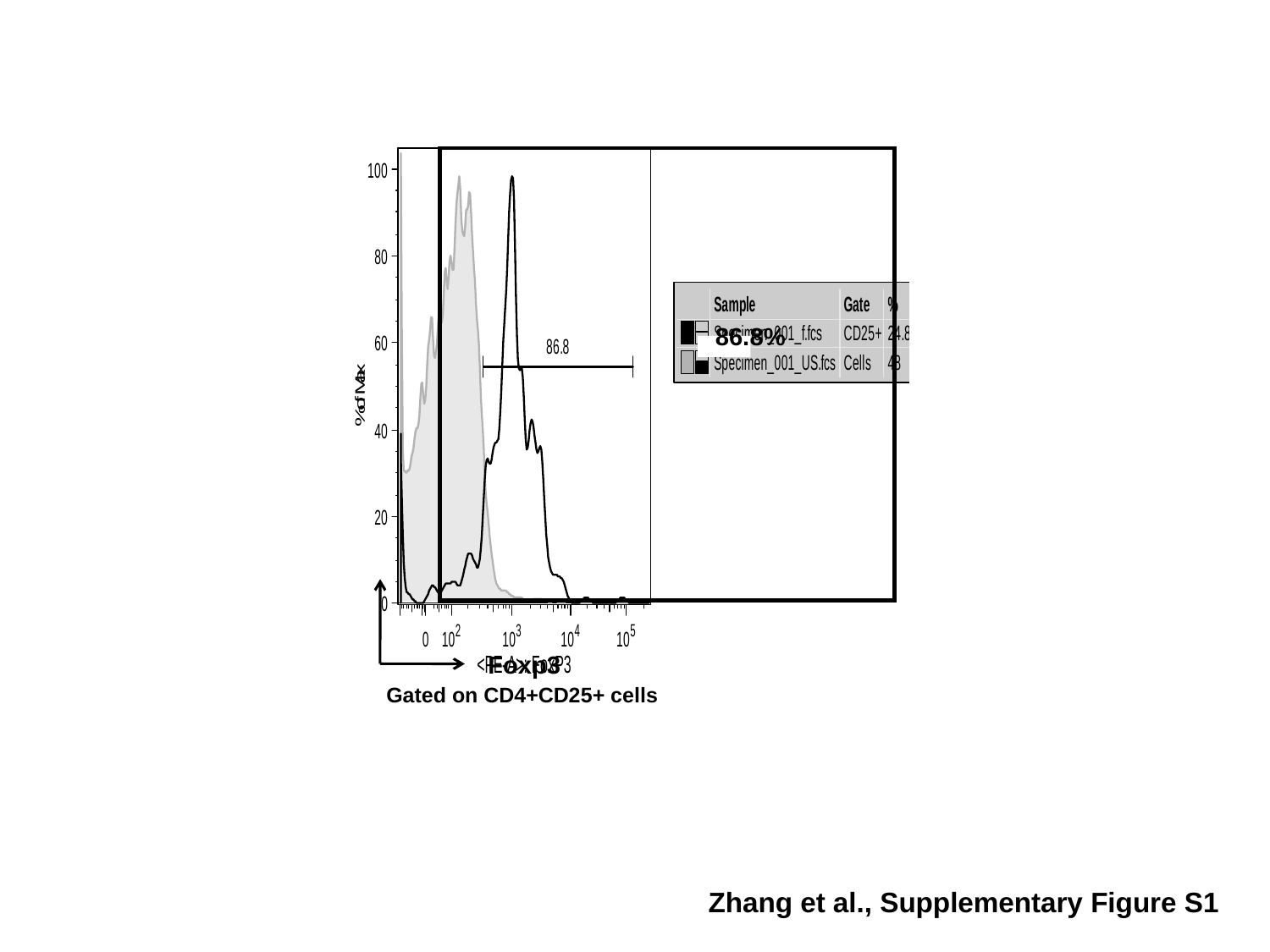

86.8%
Foxp3
Gated on CD4+CD25+ cells
Zhang et al., Supplementary Figure S1

Supplement: Figure S1 — Marker phenotypes of CD4+CD25+ Tregs. Cells were intracellularly stained with Foxp3 Abs and analyzed on gated CD4+CD25+ Tregs. Grey peak is the isotype control. The peak shifted to the right is Foxp3+ staining cells. (PPT) [file pone.0068303.s001.ppt]
