# Supplementary material for: Mesothelin Virus-Like Particle Immunization Controls Pancreatic Cancer Growth through CD8+ T Cell Induction and Reduction in the Frequency of CD4+foxp3+ICOS− Regulatory T Cells
Source: PLoS One. 2013 Jul 9;8(7):e68303. doi: 10.1371/journal.pone.0068303 (PMC3706370; doi:10.1371/journal.pone.0068303)
Supplement: Figure S2 — Characterization of Foxp3+ Treg in mice tumor tissues. Tumor tissues from PBS control group were used to characterize Foxp3+ cells in non-VLP immunized mice tumor tissues. Anti-Foxp3-PE antibody was used to stain Foxp3+ cells (red cells). Anti-CD3e-, anti-CD4-, and anti-CD8a- Abs conjugated with FITC were used to stain CD3+, CD4+, and CD8+ cells (green cells), respectively. A). CD3e+ T cell (green) and Foxp3+ cell (red) staining in tumor tissues; B). CD4+ or CD8+ T cell (green) and Foxp3+ cell (red) staining in tumor tissues. C). High power view of CD8+ T cells (green cells) and Foxp3+ T cells (red cells) localization in tumor tissues. D). At 12th day after tumor implantation and after one time VLP vaccination at day 3, mice were sacrificed for immune response analysis to compare the Foxp3+ T cell numbers in tumor tissues from three experimental groups. Results shown represents 5 different sample staining. (PPT) [file pone.0068303.s002.ppt]

## Slide 1
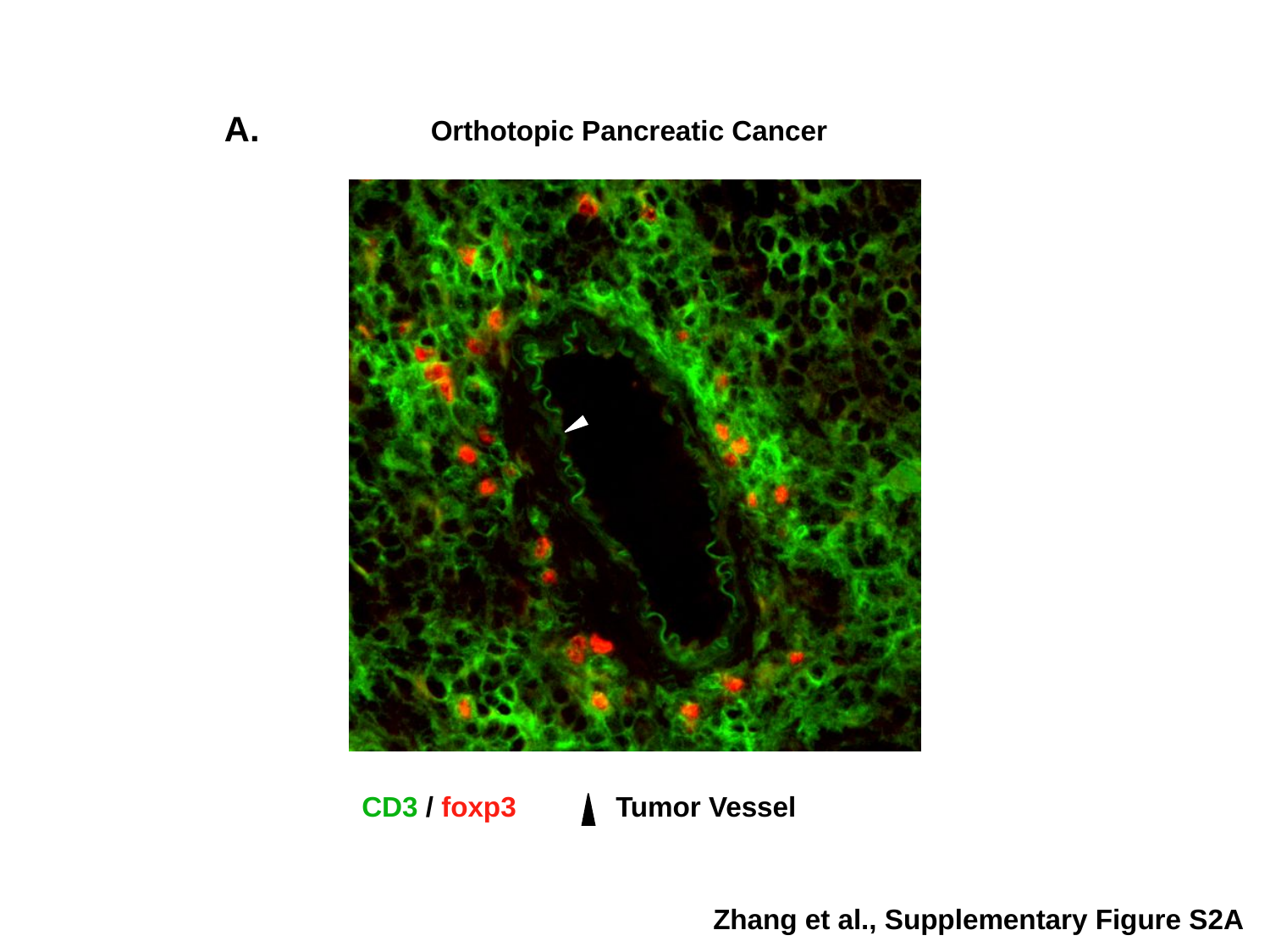

A.
Orthotopic Pancreatic Cancer
CD3 / foxp3
Tumor Vessel
Zhang et al., Supplementary Figure S2A

## Slide 2
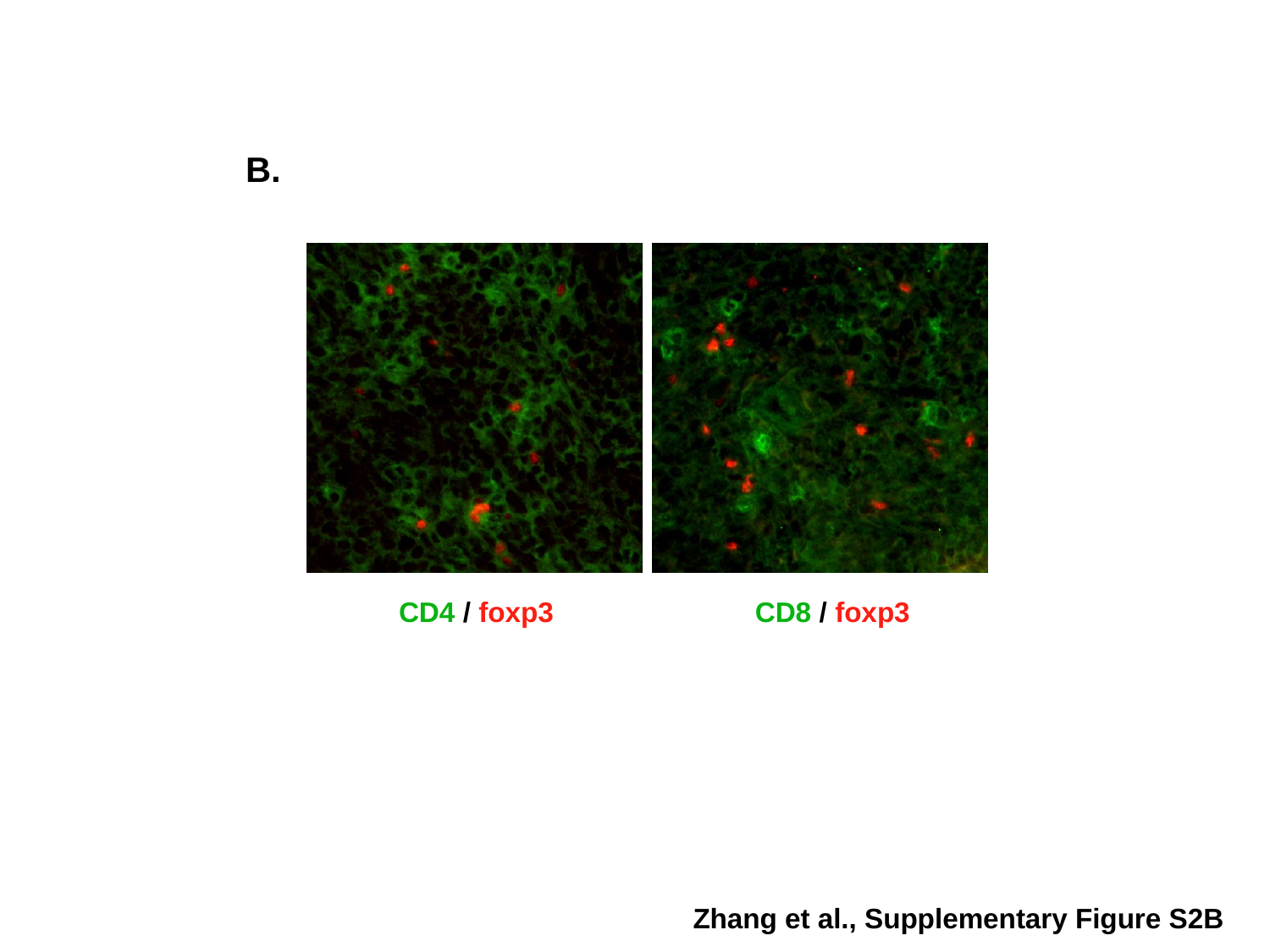

B.
CD4 / foxp3
CD8 / foxp3
Zhang et al., Supplementary Figure S2B

## Slide 3
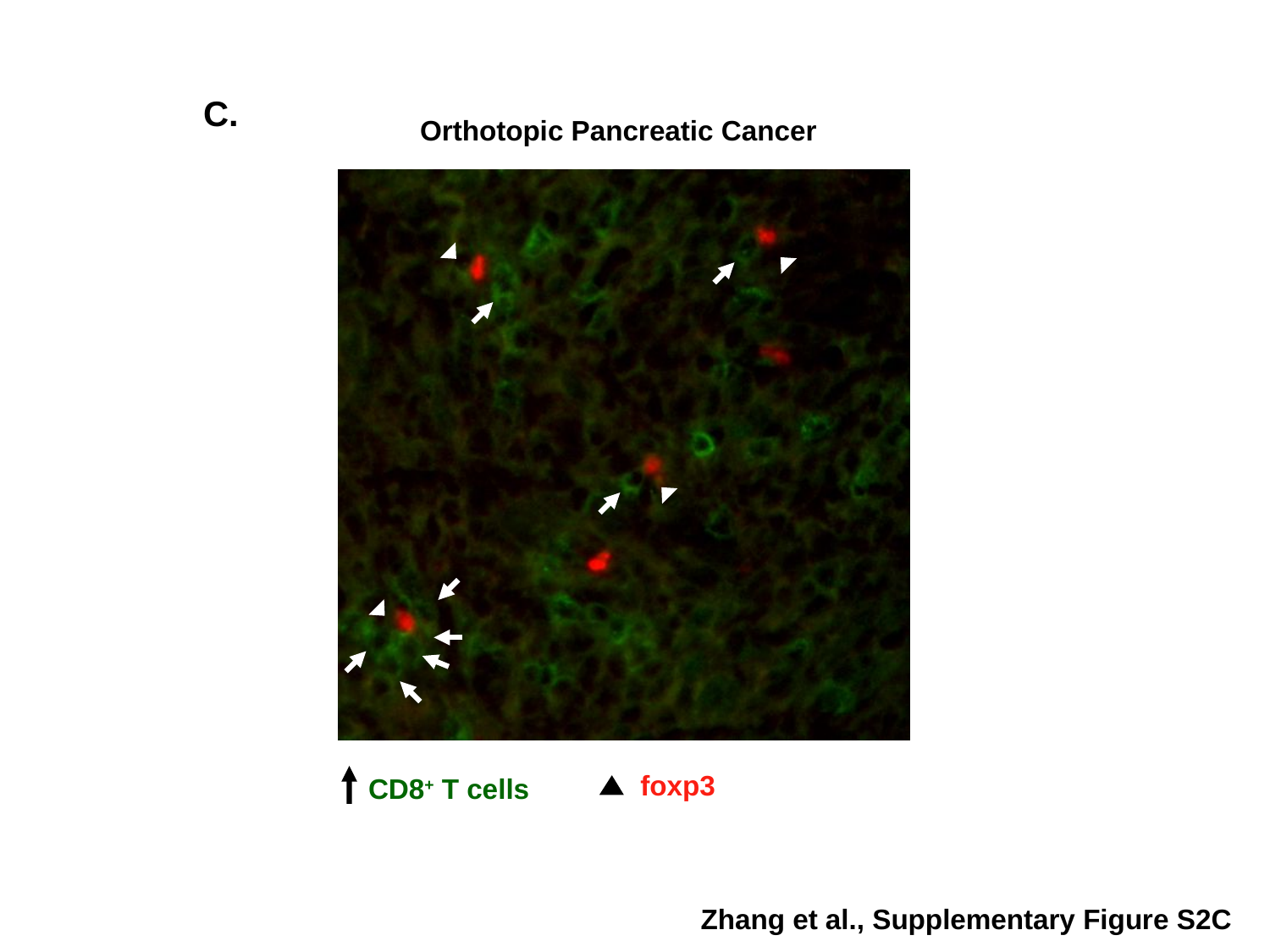

C.
Orthotopic Pancreatic Cancer
foxp3
CD8+ T cells
Zhang et al., Supplementary Figure S2C

## Slide 4
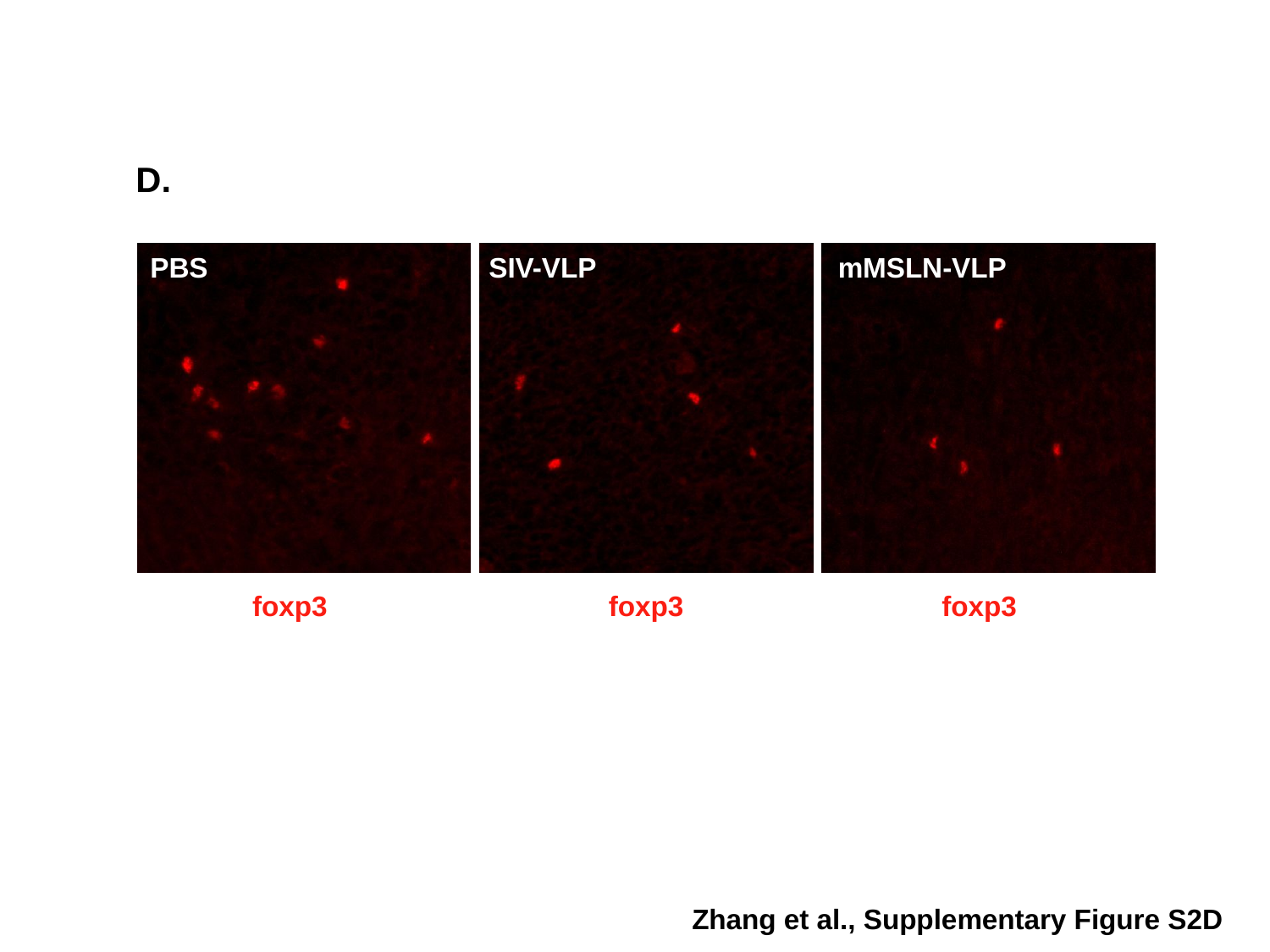

D.
PBS
SIV-VLP
mMSLN-VLP
foxp3
foxp3
foxp3
Zhang et al., Supplementary Figure S2D
