# Supplementary material for: Mesothelin Virus-Like Particle Immunization Controls Pancreatic Cancer Growth through CD8+ T Cell Induction and Reduction in the Frequency of CD4+foxp3+ICOS− Regulatory T Cells
Source: PLoS One. 2013 Jul 9;8(7):e68303. doi: 10.1371/journal.pone.0068303 (PMC3706370; doi:10.1371/journal.pone.0068303)
Supplement: Figure S3 — Characterization of Foxp3+ Treg in pancreatic cancer patient tumor tissues. Immunofluorescence staining was performed on frozen tissue blocks. A). CD3+Foxp3+ T cell staining in human PC tumor tissues. Anti-Foxp3-PE antibody was used to stain Foxp3+ cells (red cells). Anti-CD3e Ab conjugated with FITC was used to stain CD3 cells (green cells). B). Foxp3+ICOS+ T cell subpopulation staining in human tumor tissues. Anti-Foxp3-PE antibody was used to stain Foxp3+ cells (red cells). Anti-ICOS Ab conjugated with FITC was used to stain ICOS+ cells (green cells). Results shown represents 5 different sample staining. (PPT) [file pone.0068303.s003.ppt]

## Slide 1
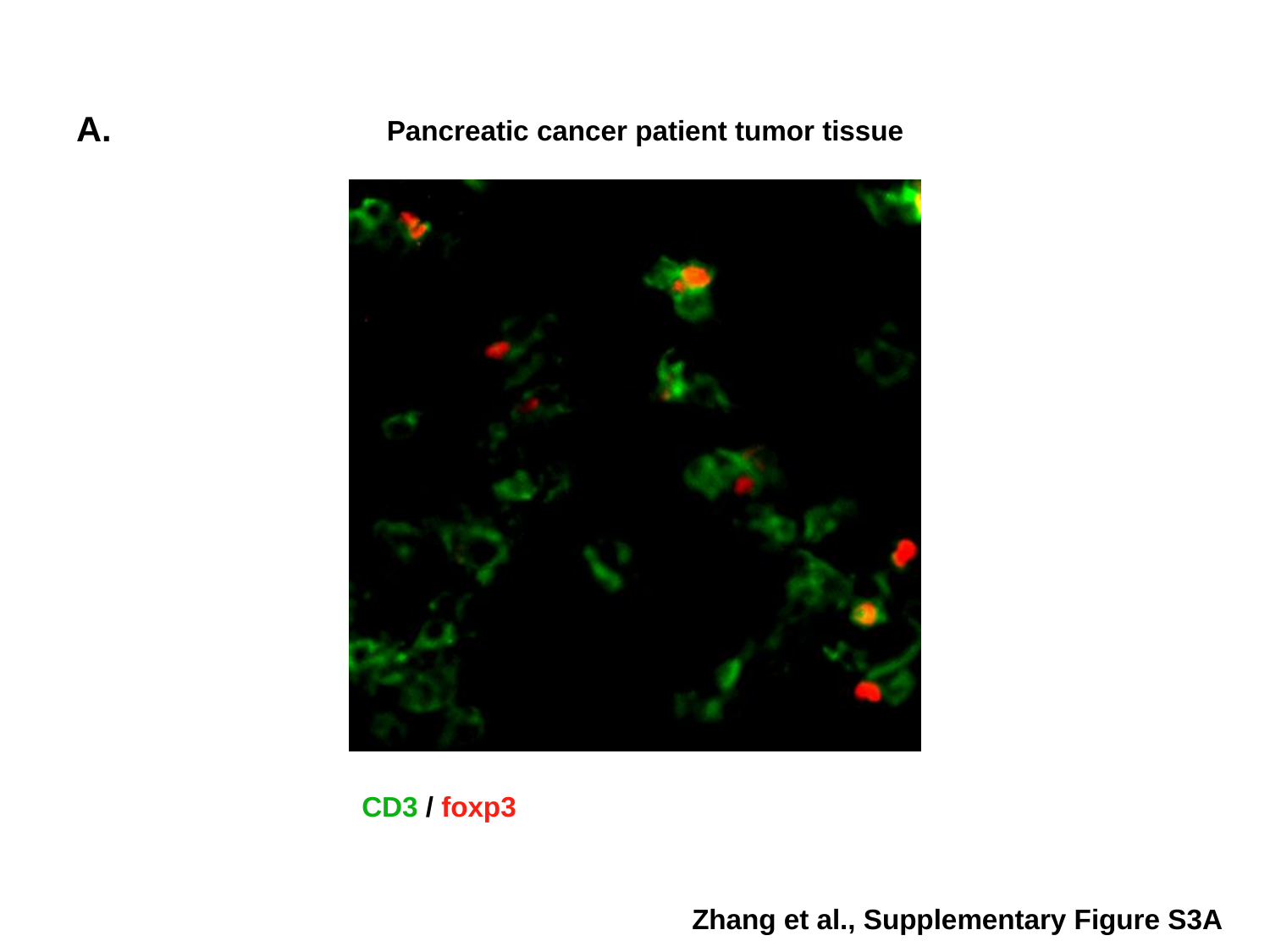

A.
Pancreatic cancer patient tumor tissue
CD3 / foxp3
Zhang et al., Supplementary Figure S3A

## Slide 2
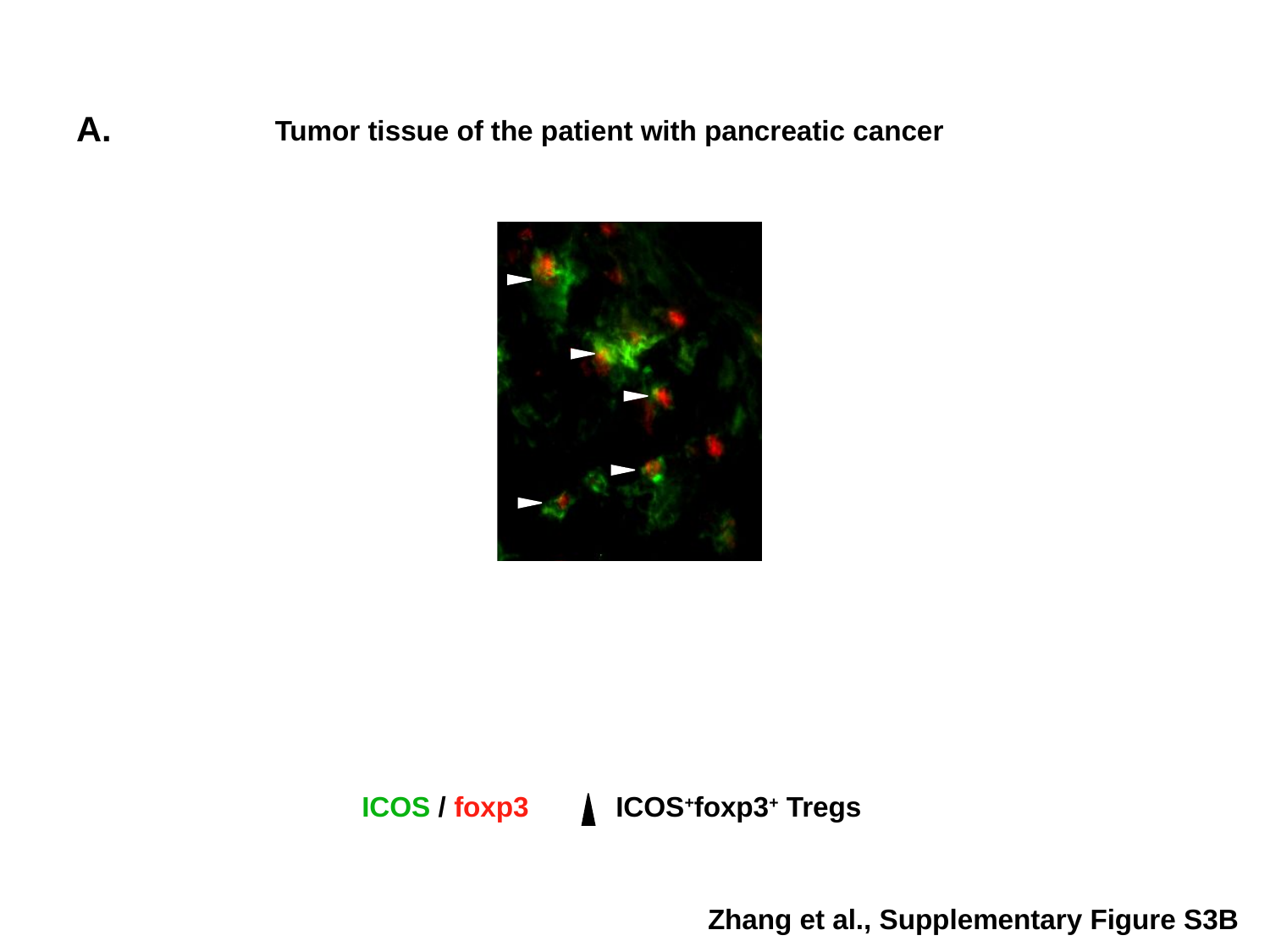

A.
Tumor tissue of the patient with pancreatic cancer
ICOS / foxp3
ICOS+foxp3+ Tregs
Zhang et al., Supplementary Figure S3B
